# Effect on bone formation of the autogenous tooth graft in the treatment of peri-implant vertical bone defects in the minipigs

**DOI:** 10.1186/s40902-015-0002-8

**Published:** 2015-01-29

**Authors:** Seok Kon Kim, Sae Woong Kim, Kyung Wook Kim

**Affiliations:** 1 Department of OMFS, College of Dentistry, Dankook University, 29 Anseodong, Cheonan, Chungnam 330-714 Korea; 2 Department of Anesthesiology and Pain Medicine, Dankook University College of Medicine, Cheonan, Korea

## Abstract

**Background:**

The aim of this study was to evaluate the effect of autogenous tooth bone as a graft material for regeneration of bone in vertical bony defects of the minipigs.

**Material and Methods:**

Six minipigs were used in this study. Four molars were extracted in the right mandibular dentition and sent to the Korea Tooth Bank for fabrication of autogenous tooth bone. Ten days later, each extraction site was implanted with MS Implant Narrow Ridge 3.0x10mm fixture (Osstem, Seoul, Korea) after standardized 2mm-sized artificial vertical bony defect formation. Pineappleshaped Root-On type autogenous tooth bones were applied to the vertical defects around the neck area of the posterior three fixtures and the fore-most one was not applied with autogenous bone as a control group. Each minipig was sacrificed at 4, 8, 12 weeks after fixture installation and examined radiologically and histologically. Histological evaluation was done under light microscope with Villanueva osteochrome bone staining with semi-quantitative histomorphometric study. Percentage of new bone over total area (NBF) and bone to implant contact (BIC) ratio were evaluated using digital software for area calculation.

**Result:**

NBF were 48.15 ± 18.02%, 45.50 ± 28.37%, and 77.13 ± 15.30% in 4, 8, and 12 weeks, respectively for experimental groups. The control group showed 37.00 ± 11.53%, 32.25 ± 26.99%, and 1.33 ± 2.31% in 4,8,12 weeks, respectively. BIC ratio were 53.08 ± 19.82%, 45.00 ± 28.37%, and 75.13 ± 16.55% in 4,8,12 weeks, respectively. Those for the control groups were 38.33 ± 6.43%, 33.50 ± 29.51 %, and 1.33 ± 2.31% in 4, 8, 12 weeks, respectively.

**Conclusion:**

Autogenous tooth bone showed higher score than control group in NBF and BIC in all the data encompassing 4,8,12 weeks specimens, but statistically significant only 12 weeks data in both NBF and BIC.

## Background

Most edentulous patients visit dental clinics only after a long time has passed after losing their teeth. Unfortunately, an appropriate recovery of oral functions is difficult in most of these patients due to accompanying alveolar bone losses. Tooth losses are unavoidably accompanied by alveolar bone losses so that satisfactory outcomes cannot be obtained in the stage of functional recovery with only the placement of implants that just substitute for teeth. The alveolar bone lost along with the lost teeth should be restored in this case. The most useful method to this end known thus far is autogenous bone grafts, which have been known to be safe in that there is no risk of foreign body reactions, as autogenous bones are immunologically the same as the alveolar bone and there is no risk of disease infection and they have excellent effectiveness in terms of absorption rates after grafts. However, methods that can substitute for autogenous bone grafts have been searched for due to autogenous bone grafts’ demerits, such as the necessity for additional surgeries for bone harvest, resulting in complications such as infection and pain, and the insufficient quantity obtained from autogenous bones in the mouth [1–3]. Recently, taking note of the fact that the constituents of teeth are similar to those of bones, methods that use teeth powder block treated through certain processes have come to the fore.

Bone grafts are being made using materials extracted from autogenous teeth. These materials have excellent biocompatibility without any immune reactions or foreign body reactions because they are autogenous tissues developed by taking note of the fact that the constituents of teeth are similar to those of bones, involve no risk of disease infection, and are not physiologically rejected by patients. When extracted teeth are demineralized, organic components are removed and the inorganic component hydroxyapatite remains as a main component. The components of tooth bone, which is a graft material made by using teeth, have already been experimentally verified in many studies and papers [4–9]. However, those studies are different from the present study, as most of them are in regard to tooth bone made from other persons’ teeth already extracted after removing all organic components for immunological safety and safety against infection transmission. The present study approached the effectiveness of autogenous tooth bone grafts that have the same benefits as autogenous bone grafts in that rejection of transplant can be fundamentally blocked because autogenous tooth bone, immunologically speaking, has the same origin as autogenous bone, so that the possibility of the transmission of a source of infection between different persons or species can be fundamentally blocked.

In the present study, the effects of bone graft materials made using extracted autogenous teeth were examined through animal experiments using minipigs. Vertical bone defects were artificially made to control the amount of lost bones, autogenous tooth bone prepared in advance was grafted together with implants, and the histological findings were observed to examine bone formation effects.

## Methods

### Study materials

#### Experimental animals

In the present experiment, a total of six healthy male minipigs raised under the same conditions for a certain period (approximately 35-40 kg, 24 months old, Prestige World Genetics, Pyeongtaek, Korea) were used as experimental animals.

#### Graft material

Autogenous tooth bone graft materials made from teeth extracted from the above minipigs were grafted on individual minipigs after placing implants (MS Implant Narrow Ridge 3.0×10 mm, OSSTEM).

### Study method

#### Animal experiment

**Figure 1:**
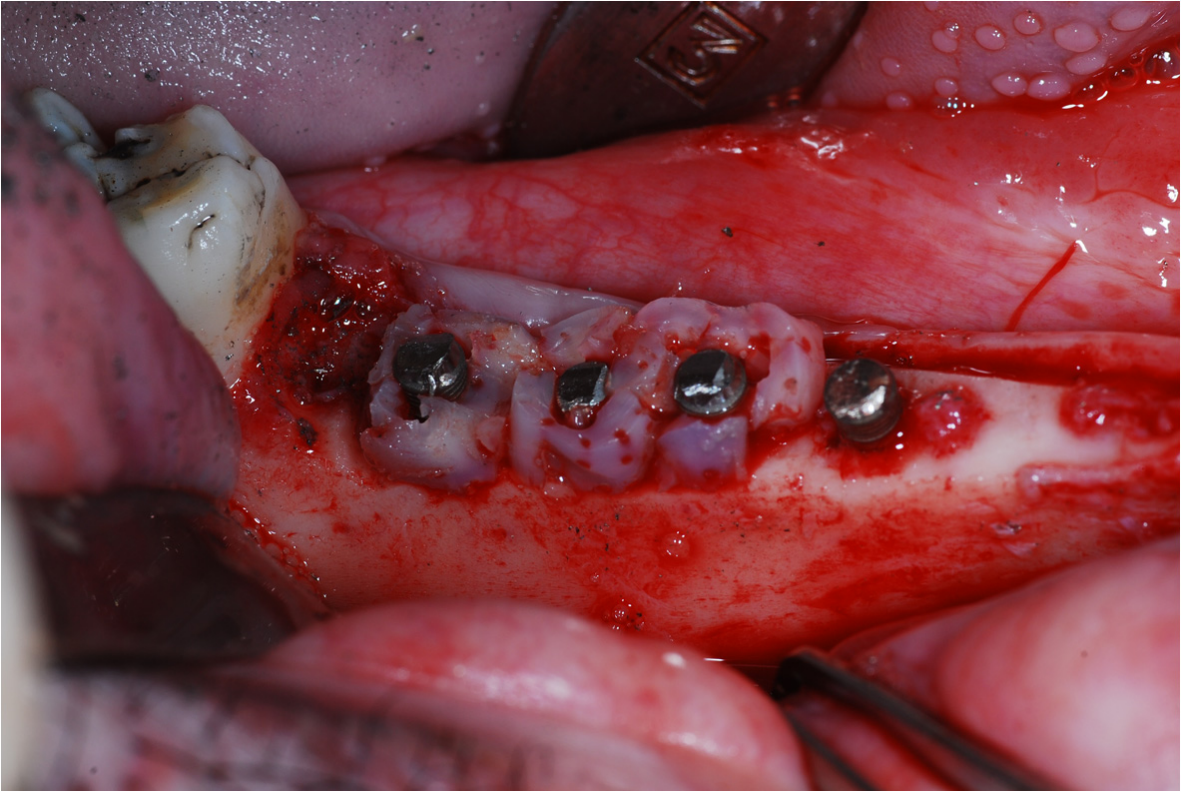
**Pineapple-shaped root on type autogenous tooth bones were applied to the vertical defects around the neck area of the posterior three fixtures and the foremost one was not applied with autogenous bone as a control group.**

Each of the selected minipigs was pretreated with an intravascular injection (IV) of Atropin (Kwangmyung Pharmaceutical Ind. Co. Ltd., Seoul, Korea) and intramuscular injections of xylazine (Rompun, Bayer Korea Co. Seoul, Korea) and ketamin (Ketara, Yuhan Co., Seoul, Korea) and was put under anesthesia throughinhalation anesthesia using enflurane (Gerolan, Choongwae Pharmaceutical Co., Seoul, Korea). Infiltration anesthesia was conducted on the surgical site using lidocaine (2% lidocaine hydrochloride - epinephrine, 1.8 ml, Yuhan Co., Seoul, Korea) for anesthesia and hemostasis. Four molar teeth on the right side of the lower jar were extracted and processed into autogenous tooth bone. On day 10 after tooth extraction, a buccolingual valve was formed and lifted, 2 mm deep vertical bone defects were formed in the four extraction sockets, and four implants (MS Implant Narrow Ridge 3.0×10 mm, OSSTEM) were placed in the extraction sockets. The foremost implant was not grafted with the autogenous tooth bone so that it could be used as a control group and the remaining rear implants were grafted with the autogenous tooth bone as an experimental group. The grafted autogenous tooth bone was grafted in the form of a processed pineapple shape that surrounded the peri-implant vertical bone defects. (Figure 1) Thereafter, the valve was pulled into its place and sutured using an absorbable surgical suture (3/0 Vicryl). Immediately after the surgery, an antibiotic (Penicillin G Sodium, Woojin B&G, Korea, 1 ml/10 kg, 1 time/2 days) was intramuscularly injected and the minipig was made to take liquid food until macroscopic wound healing was completed to protect the surgical site.

#### Fabrication of autogenous tooth bone

Extracted teeth immersed in 70% ethyl alcohol were sent to a specialized processing institution (Korea Tooth Bank Co., Seoul, Korea), made into block shapes after attached soft tissues and foreign substances such as tartar were removed, put into a distilled water and hydrogen oxide solution, and cleaned with an ultrasonic cleaner to remove remaining foreign substances. The cleaned particles were dehydrated using ethyl alcohol and underwent a defatting process using an ethyl ether solution. The autogenous teeth that underwent these processes were made to undergo a lyophilization process, disinfected using ethylene oxide gas, packed and delivered to the laboratory, and used in graft processes.

#### Fabrication and analysis of tissue specimens

Before fabricating specimens for tissue observation from the experimental animals, a mixture of xylazine HCL (2.3 mg/kg, Bayer) and ketamine (5 mg/kg, Yuhan Corporation) was intravenously injected into the minipigs at four, eight, and 12 weeks after graft to put the minipigs under anesthesia. Then, KCL (2 mmol/1 g, Huons) was swiftly injected into the veins to administer euthanasia and bone fragments including implements and adjacent tissues were collected immediately after the sacrifice.

The tissues collected from two minipigs per each week of collection were used in histomorphometric analysis. The collected tissues were fixed in 70% alcohol for three days and then stained first by leaving them in a Villanueva staining solution for seven to10 days. For dehydration and bleaching, the tissues were left in each of 50, 70, 80, 95, and 100% alcohol solutions for four hours and then left in propylene oxide overnight for complete dehydration. Blocks were made using epoxy resin (Eponate, Ted Pella Inc., Redding, CA, USA) and hardened for three days in an incubator at 60°C. Thereafter, trimmed sections were made using an Accutom-50 (Struers Co., Copenhagen, Denmark) and the trimmed sections were ground using a micro-cutting and grinding system (EXAKT, Exakt Co., Norderstedt, Germany) to make 10-20 μm thick tissue slides.

#### Histomorphometric analysis

Images magnified by factors of 12.5 and 40 were obtained using an optical microscope (Axioscop, Carl Zeiss, Jena, Germany) and the images were reconstructed to photos of the entire tissues using the Photoshop program. From these photos, the ratio of newly formed mineralized bones (NBF, new bone formation) to the entire bone defect was calculated using an image analyzer (iMTechnology, Korea). The area of newly formed bones was regarded as the area to newly formed bone boundaries including mineralized bones, remaining bone graft materials, bone marrow, fibrous connective tissues, and newly formed blood vessels. In addition, the total length of bone defects in each implant was measured as well as the bone areas in contact with the implant to calculate the contact ratio between the bones and the implant. (Villanueva stain, original magnification ×12.5, magnified photo Villanueva stain, original magnification ×40).

As a statistical method, independent sample t-tests were conducted using SSPS ver.17.0 (SPSS, Chicago, IL, USA) and cases where the P value was smaller than 0.05 were regarded as being statistically significant.

#### Comparison and evaluation of peri-implant bone densities in panoramas

**Figure 2:**
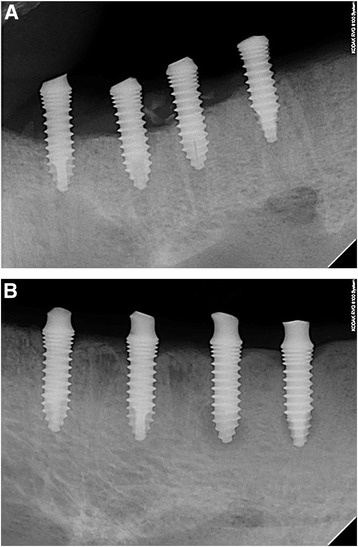
**Distal three implants were treated with autogenous tooth bone and showed more radio-opacity around implant than foremost implant after 12 weeks. A**. 4 weeks panoramic view. **B**. 12 weeks panoramic view.

To compare and evaluate bone densities, panorama photos (DP-80-P/PM2002 EC Proline, Finland) were taken from the control group that was not treated at all after implant placement and the experimental group grafted with bone after implant placement at four weeks and 12 weeks after implant placement (Figure 2). The panorama photos, digitized as DICOM files, were converted into JPEG graphic files, and the JPEGs were stored. From the stored files, bone densities were measured in the area around each implant ranging from 2 mm in front of the implant boundary to 2 mm in the rear of the implant boundary and to 2 mm downward from the upper bone boundary considering the size of the autogenous tooth bone graft material using the average value of the 255 tonality grayscale using the gray-level histogram of the Adobe Photoshop CS3 program. The peri-implant bone densities were measured in panoramas using the Adobe Photoshop CS3 program.

As a statistical method, the non-parametric Wilcoxon test of the SPSS 12.0 program (SPSS Inc. Chicago, USA) was used, and the statistical significance level was set to P < 0.05.

#### Comparison and evaluation of peri-implant bone densities on CT

To compare and evaluate bone densities, CT (DCTP-110-p/AlphardVEGA-3030, Asahi Roentgen Ind. Co., Japan) imaging was conducted with the control group and the experimental group at 12 weeks after implant placement. Thereafter, the photos digitized as DICOM files were converted into JPEG graphic files and the JPEGs were stored. From the stored files, bone densities were measured in the area around each implant of the minipigs (ranging from 2 mm in the buccolingual direction to 2 mm downward from the lingual upper bone boundary) using the average value of the 255 tonality gray scale using the gray-level histogram of the Adobe Photoshop CS3 program. A measurement was conducted on the axial view. The non-parametric Mann–Whitney test of the SPSS 12.0 program (SPSS Inc. Chicago, USA) was used and the statistical significance level was set to P < 0.05.

## Results

### Histomorphometric findings

#### Histological findings

**Figure 3:**
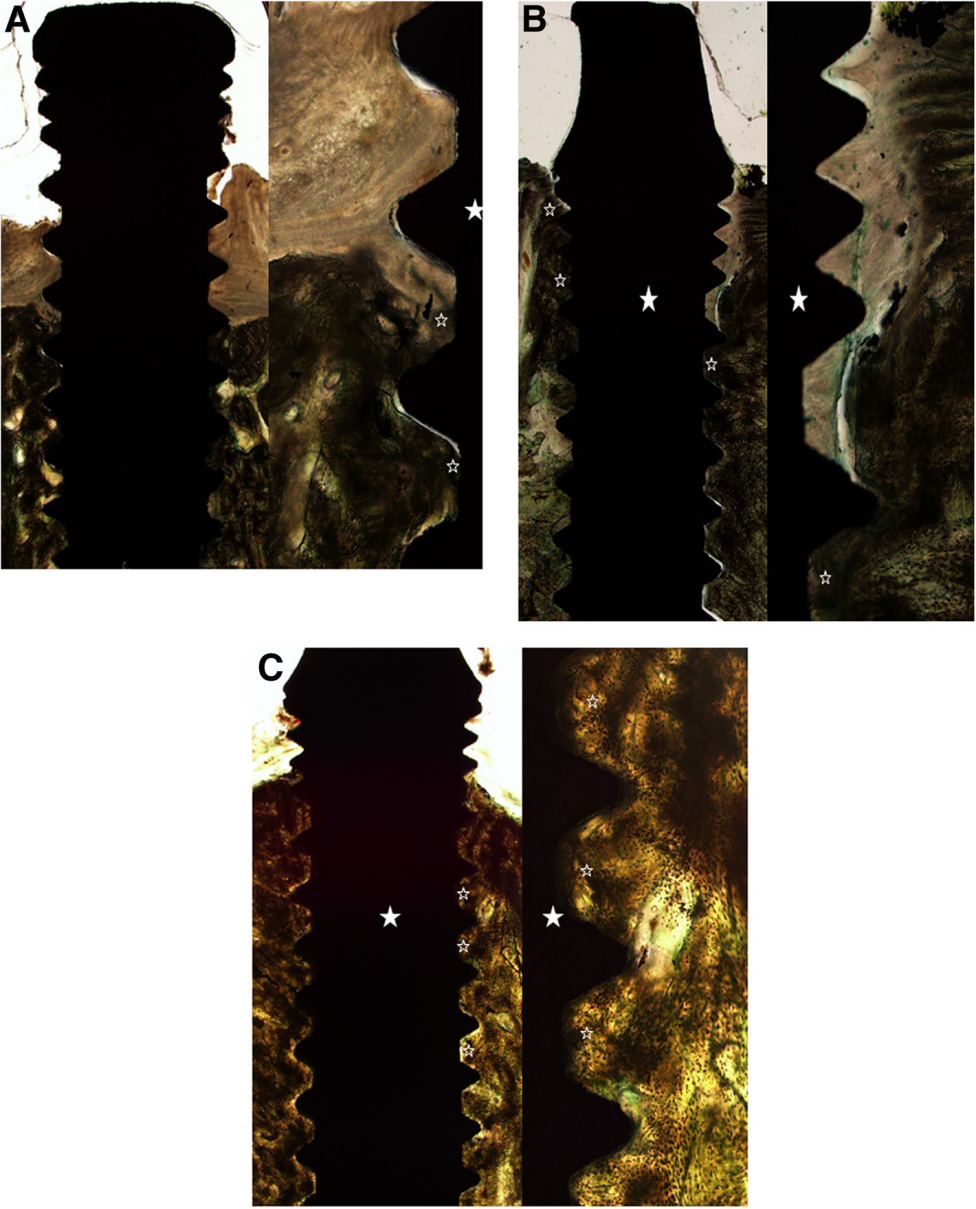
**Microphotographs of experimental group. A**. Histopathologic findings in 4 weeks, Lt: Low power view showed increased new bone formation (open asterisks) and bone-implant contact(BIC) ratio around the implant (asterisk). Rt: Higher power view of the left lower half of the left figure showed higher new bone formation (open asterisks) and BIC around the implant (asterisk). Lt: x12.5, Rt:x40. Villanueva osteochrome bone stain. **B**. Histopathologic findings in 8 weeks, Lt: Low power view showed increased new bone formation (open asterisks) and bone-implant contact(BIC) ratio around the implant (asterisk). Rt: Higher power view of the right upper one third of the left figure showed higher new bone formation (open asterisks) and BIC around the implant (asterisk). Lt:x12.5, Rt:x40. Villanueva osteochrome bone stain. **C**. Histopathologic findings in 12 weeks, Lt: Low power view showed significantly increased new bone formation (open asterisks) and bone-implant contact(BIC) ratio around the implant(asterisk). Rt: Higher power view of the right upper half of the left figure showed higher new bone formation (open asterisks) and BIC around the implant (asterisk). Lt:x12.5, Rt:x40. Villanueva osteochrome bone stain.

**Figure 4:**
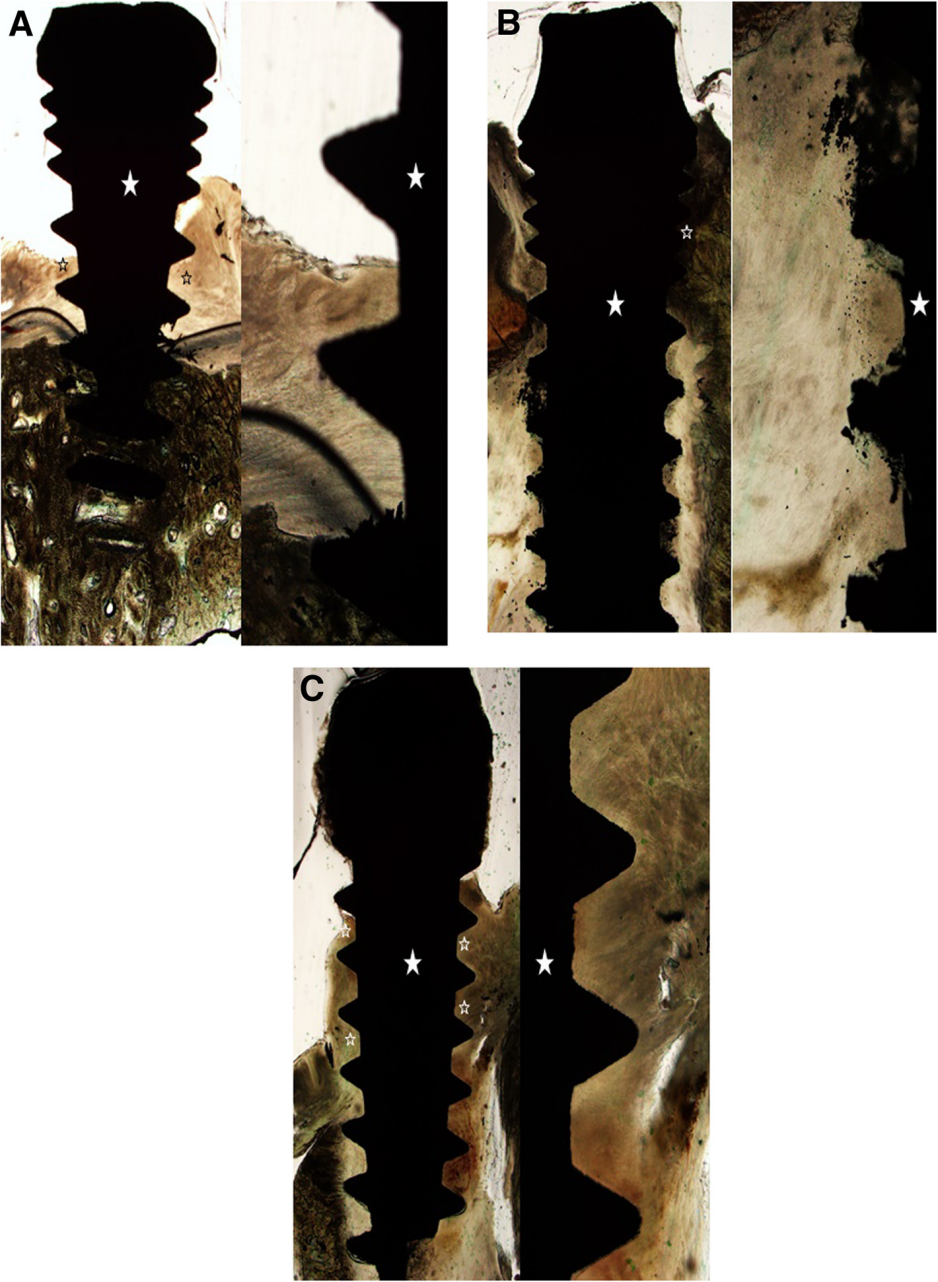
**Microphotographs of control group. A**. Histopathologic findings in 4 weeks, Lt: Low power view showed little new bone formation (open asterisks) around the implant (asterisk). Rt: Higher power view of the left midportion of the left figure showed absence of new bone formation around the implant (asterisk). Lt:x12.5, Rt:x40. Villanueva osteochrome bone stain. **B**. Histopathologic findings in 8 weeks, Lt: Low power view showed little new bone formation (open asterisks) around the implant (asterisk). Rt: Higher power view of the left lower half of the left figure showed absence of new bone formation around the implant (asterisk). Lt:x12.5, Rt:x40. Villanueva osteochrome bone stain. **C**. Histopathologic findings in 12 weeks, Lt: Low power view showed little new bone formation (open asterisks) around the implant (asterisk). Rt: Higher power view of the right midportion of the left figure showed absence of new bone formation around the implant (asterisk). Lt:x12.5, Rt:x40. Villanueva osteochrome bone stain.

After the bone graft, the experimental group showed an aspect of active new bone formation and grafted bone absorption and replacement by newly formed bones through substitution were observed. New bone formation was observed from the margin of the defect toward the center of the defect (Figure 3). Although the control group also showed an aspect of new bone formation, the degree was lower compared to the experimental group (Figure 4). Although the above-mentioned aspects were observed in findings at weeks four, eight, and 12, statistically significant results could only be obtained from the week 12 groups.

#### Ratio of newly formed mineralized bones (NBF, new bone formation) to entire defects

According to the results of histomorphometric analysis, the amount of newly formed bones was larger in the experimental group compared to the control group. The newly formed bone area ratios of the experimental group at weeks four, eight, and 12 (Figure 3) were 48.15 ± 18.02%, 45.50 ± 28.37%, and 77.13 ± 15.30%, respectively, which were higher than those of the control group (Figure 4), which were 37.00 ± 11.53%, 32.25 ± 26.99%, and 1.33 ± 2.31%, respectively, (Table 1) and the difference between the week 12 groups was statistically significant (Table 2).

**Table 1:** **Results for new bone formation area (%)**

	**Control**	**Experimental**
4 weeks	37.00 ± 11.53	48.15 ± 18.02
8 weeks	32.25 ± 26.99	45.50 ± 28.37
12 weeks	1.33 ± 2.31	77.13 ± 15.30

**Table 2:** **Significant difference between the control and experimental group in NBF rate**

	**Control**	**Experimental**
4 weeks	-	-
8 weeks	-	-
12 weeks	-	*

*Statistically significant difference relative to group 1 in 12 weeks: P < 0.05.

### Bone to implant contact ratio (BIC)

According to the results of histomorphometric analysis, bone-to-implant contact ratios were also higher in the experimental group compared to the control group. The newly formed bone area ratios of the experimental group at weeks four, eight, and 12 were 53.08 ± 19.82%, 45.00 ± 28.37%, and 75.13 ± 16.55%, respectively, which were larger than those of the control group, which were 38.33 ± 6.43%, 33.50 ± 29.51%, and 1.33 ± 2.31%, respectively (Table 3). Again, the week 12 groups showed a statistically significant difference (Table 4).

**Table 3:** **Results for bone to implant contact ratio (%)**

	**Control**	**Experimental**
4 weeks	38.33 ± 6.43	53.08 ± 19.82
8 weeks	33.50 ± 29.51	45.00 ± 28.37
12 weeks	1.33 ± 2.31	75.13 ± 16.55

**Table 4:** **Significant difference between the control and experimental group in BIC ratio**

	**Control**	**Experimental**
4 weeks	-	-
8 weeks	-	-
12 weeks	-	†

†Statistically significant difference relative to group 1 in 12 weeks: P < 0.05.

### Results of comparison and evaluation of peri-implant bone densities in panoramas

According to the results of comparison of changes in peri-implant bone densities of the control group and the experimental group in panorama photos at weeks four and 12 after implant placement, the control group showed average 7 gray-level scale increases, while the experimental group showed average 10 gray-level scale increases, which were larger than those of the control group, but the bone density increases over time in both the control group and the experimental group were significant (p < 0.05) (Figure 2, Table 5).

**Table 5:** **Bone density increase evaluation at four weeks and 12 weeks in panoramic view**

	**4 weeks bone density**	**12 weeks bone density**	
	**Mean ± SD**	**Mean ± SD**	**p-value**
Control group	71 ± 15.97	78 ± 19.23	.027
Experimental group	88 ± 16.34	98 ± 14.23	.027

### Comparison and evaluation of peri-implant bone densities on CT

**Figure 5:**
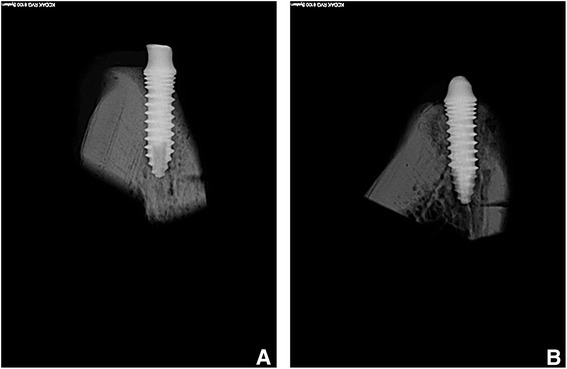
**Control group showed less radio-opacity than subject group after 12 weeks. A**. Control group. **B**. Experimental group.

From the CT images taken from the implants of minipigs sacrificed at week 12 for comparison of bone densities, the average value of the control group was measured as 87 gray level scale on average and that of the experimental group was measured as 128 gray level scale on average, which was higher by 41 gray-level scale levels on average than that of the control group. The bone densities of the experimental group in CT images were shown to be higher compared to the control group, and the differences were statistically significant (p < 0.05) (Figure 5, Table 6).

**Table 6:** **Bone density comparative evaluation after 12 weeks in CT view**

		**Control group**	**Experimental group**	
		**Mean ± SD**	**Mean ± SD**	**p-value**
Bone density after 12 weeks	Gray scale	87 ± 35.14	128 ± 30.61	.037

After the bone graft, the experimental group showed an aspect of active new bone formation and grafted bone absorption and replacement by newly formed bones through substitution were observed. New bone formation was observed from the margin of the defect toward the center of the defect (Figure 3). Although the control group also showed an aspect of new bone formation, the degree was lower compared to the experimental group (Figure 4). Although the above-mentioned aspects were observed in findings at weeks four, eight, and 12, statistically significant results could only be obtained from the week 12 groups. In addition, according to findings from panorama and CT images, it could be seen that increases in bone densities in that experimental group were larger than those in the control group. The differences were statistically significant.

## Discussion

After the fact that demineralized bones have heterotopic osteogenicity was revealed by Urist [10], the same group reported that demineralized teeth have the same property [11]. Thereafter, despite content in textbooks that said the constituents of teeth are similar to those of bones, the idea of using demineralized teeth as a bone graft material was not actually applied to clinical areas until a long time had passed and the idea was recently reported by domestic research teams to the academic world [12], leading to a sudden increase in attention. Autogenous tooth bones are quite likely to be utilized as a good substitute material for autogenous bones because they have an advantage of being immunologically equivalent to autogenous bones because they are bones generated from autogenous teeth as well as involving no onerousness such as the additional operations in donor sites required when autogenous bones are grafted. Autogenous tooth bone grafts have been already introduced into clinics and have been showing good outcomes in radiological and histological tests [13,14].

In the present study, minipigs’ teeth were extracted and after undergoing a 10 day fabricating period, artificial vertical bone defects were immediately formed and processed pineapple-shaped autogenous tooth bone were grafted together with implant placing bodies in a form surrounding the implant placing bodies. A healing group provided with no treatment so that artificial bone defects would be naturally healed was selected as a control group to experiment on the effects of autogenous tooth bone blocks. Although there is a time difference of 10 days, this experiment minimized the fabrication period to differentiate the experimental group and the control group in the shortest possible period, and these authors are confident that this is the most accurate experiment on the effects of autogenous tooth bone. Given that this was not an experiment to form bone defects, wait until the wounds are healed, and graft bones onto chronic bony defects, this experiment has a limitation in that it could not consider the characteristics of existing chronic diseases such as periodontal diseases. However, bearing this situation in mind, the fact that if some time is allowed to pass after extraction, experiments cannot be standardized as the shapes of vertical bone defects cannot be controlled was considered.

Whereas past studies on bone graft materials using teeth removed all of the organic components because of the risks of immunological foreign body reactions and infection, the present study was free of such risks, as the bone graft material was autogenous teeth, and thus organic matter could be preserved intact. Therefore, in addition to the bone conduction effect that is expected from existing synthetic bones made using allogenic bones, heterogeneous bones, etc., the bone-inducing effects of growth factors consisting of such organic matter could be also expected, and this fact is an advantage of autogenous tooth bone graft materials being differentiated from existing bone graft material. In fact, according to the results of an animal experiment that histomorphometrically studied autogenous tooth bones in comparison with pure synthetic bone products in minipigs, the ratio of newly formed bones was higher when autogenous tooth bones were used than when synthetic bones were used [15].

According to an experimental paper written by Tomoki, et al. [16] that compared the effects of autogenous tooth graft materials from mice and the effects of autogenous bones collected from mice’s iliac bone in mice’s lower jaw bone defects, autogenous tooth graft materials show more excellent bone formation than did the autogenous bones collected from mice’s iliac bone. The present experiment used minipigs, because they are one of the animals frequently used in bone graft experiments in the dental area as the basic constituents of minipigs’ alveolar bone are similar to those of humans, and they are one of the animals frequently used in studies of stem cells and bone graft experiments in the dental area for many reasons [17]. In previous studies that histomorphometrically observed the effects of autogenous tooth bones in comparison with synthetic bones as a control group in existing lower jaw defects of minipigs, new bone formation was also more excellent in the experimental group [15]. In the present study, the fact that autogenous tooth bones were used in vertical bone augmentation after forming artificial vertical bone defects in the lower jaws of minipigs is meaningful because it is a verification of block-shaped graft materials that are effective not only in bone defects with good bony housing but also vertical bone defects with poor prognoses. Following the present study, conducting studies of the effects of autogenous tooth bones in comparison with autogenous bones using minipigs is thought to be a good future research task.

When teeth have been lost, the alveolar bone begins disuse involution because it cannot be mechanically stimulated by masticatory force anymore [18]. When the alveolar bone has been lost along with teeth losses, previous forms cannot be anatomically reproduced because the shape of the site where teeth existed previously is changed. When alveolar bone involution progresses, it will eventually accompany the form of vertical bone defects, and this situation can be encountered in most patients that visit dental clinics. In particular, compared to those forms of defects that maintain bone shapes, in the case of vertical bone defects accompanied by height decreases, poor prognoses are found when observed for long periods of time after bone grafts in most cases [19]. Although the shapes of bone grafts can be divided into many types, the reason why the shape of a block was selected was that vertical bone defects correspond to zero wall bone defects that lack bony housing that can maintain its shape. Since powder graft materials cannot maintain their shapes, when there are vertical bone defects, bone grafts should be performed in the form of blocks that can maintain their shapes. The processed pineapple shape used in the present experiment has excellent effects in the reproduction of the form of vertical bone defects around implant placing bodies, and the selection of block-shaped graft bones avoiding powder-shaped ones became another factor contributing to the successful outcomes.

Despite its limitations, such as the fact that the results of non-parametric statistical processing were not statistically significant because the number of experimental animals used was small, the fact that the present study was not a long-term follow-up study but was a fragmentary cross-sectional study, and the fact that the present experiment was not an experiment to form bone defects, wait until the wounds are healed, and graft bones onto chronic bony defects, the present study can be said to be meaningful in that it is the first animal experiment conducted with minipigs that verified the effects of autogenous tooth bone graft materials in relation to vertical bone defects. Although additional experimental studies are necessary to evaluate the bone-inducing ability of autogenous tooth bone graft materials in more depth, based on the related information known thus far, autogenous tooth bone grafts that can maintain the advantages of autogenous bone grafts and can supplement the shortcomings of autogenous bone grafts and damage to donor sites are thought to be a new bone graft method that can substitute for autogenous bone grafts.

## Conclusion

The purpose of the present study was to make bone graft materials using extracted autogenous teeth and examine the effects of the bone graft materials when they have been grafted onto artificially formed vertical bone defects together with implant placement. According to the results of the experiment, the experimental group showed larger bone formation effects than the control group at all of weeks four, eight, and 12. Among the differences, those at week 12 were statistically significant.

1. At all of weeks four, eight, and 12, the experimental group grafted with autogenous tooth graft materials showed higher BIC and NBF compared to the control group. However, the differences at weeks four and eight were not statistically significant.
2. At week 12 after implant placement, the experimental group grafted with autogenous tooth graft materials showed statistically significantly higher BIC and NBF compared to the control group (p < 0.05).
3. In a comparison of bone densities in panoramas at weeks four and 12, although the experimental group showed more increases in bone densities, both the experimental group and the control group showed significant increases over time (p < 0.05).
4. In CT findings, the experimental group showed higher bone densities compared to the control group at week 12 and the difference was significant (p < 0.05).
5. Based on the above results, autogenous tooth bone graft materials are thought to be good graft materials that can substitute for existing autogenous bone graft materials.
